# Dejerine–Sottas disease in childhood—Genetic and sonographic heterogeneity

**DOI:** 10.1002/brb3.919

**Published:** 2018-02-21

**Authors:** Sanne M. R. Hobbelink, Cain R. Brockley, Rachel A. Kennedy, Kate Carroll, Katy de Valle, Padma Rao, Mark R. Davis, Nigel G. Laing, Nicol C. Voermans, Monique M. Ryan, Eppie M. Yiu

**Affiliations:** ^1^ Neurology Department Radboud University Medical Center Nijmegen The Netherlands; ^2^ Medical Imaging Department The Royal Children's Hospital Melbourne Parkville Vic. Australia; ^3^ Neurology Department The Royal Children's Hospital Melbourne Parkville Vic. Australia; ^4^ Neurosciences Research Murdoch Childrens Research Institute Parkville Vic. Australia; ^5^ Neurogenetics Unit Department of Diagnostic Genomics PathWest Laboratory Medicine QEII Medical Centre Nedlands WA Australia; ^6^ QEII Medical Centre Centre for Medical Research University of Western Australia and Harry Perkins Institute of Medical Research Nedlands WA Australia; ^7^ Department of Paediatrics The University of Melbourne Parkville Vic. Australia

**Keywords:** Charcot–Marie–Tooth disease, Dejerine–Sottas disease, pediatric, peripheral neuropathy, ultrasound

## Abstract

**Introduction:**

The nerve sonographic features of Dejerine‐Sottas disease (DSD) have not previously been described.

**Methods:**

This exploratory cross‐sectional, matched, case–control study investigated differences in nerve cross‐sectional area (CSA) in children with DSD compared to healthy controls and children with Charcot–Marie–Tooth disease type 1A (CMT1A). CSA of the median, ulnar, tibial, and sural nerves was measured by peripheral nerve ultrasound. The mean difference in CSA between children with DSD, controls, and CMT1A was determined individually and within each group.

**Results:**

Five children with DSD and five age‐ and sex‐matched controls were enrolled. Data from five age‐matched children with CMT1A was also included. Group comparison showed no mean difference in nerve CSA between children with DSD and controls. Individual analysis of each DSD patient with their matched control indicated an increase in nerve CSA in three of the five children. The largest increase was observed in a child with a heterozygous *PMP22* point mutation (nerve CSA fivefold larger than a control and twofold larger than a child with CMT1A). Nerve CSA was moderately increased in two children—one with a heterozygous mutation in *MPZ* and the other of unknown genetic etiology.

**Conclusions:**

Changes in nerve CSA on ultrasonography in children with DSD differ according to the underlying genetic etiology, confirming the variation in underlying pathobiologic processes and downstream morphological abnormalities of DSD subtypes. Nerve ultrasound may assist in the clinical phenotyping of DSD and act as an adjunct to known distinctive clinical and neurophysiologic findings of DSD subtypes. Larger studies in DSD cohorts are required to confirm these findings.

## 1 INTRODUCTION

Dejerine–Sottas disease (DSD) comprises a genetically heterogeneous group of early‐onset demyelinating hereditary neuropathies. The term was initially used to describe a clinical phenotype characterized by symptom onset in the first two years of life, delayed motor development, hypotonia, and extremely slow nerve conduction (median motor nerve conduction velocity, MNCV, of 12 m/s or less; Gabreels‐Festen, 2002; Ouvrier, McLeod, & Conchin, 1987; Yiu & Ryan, 2012). Other common clinical features include areflexia, muscle wasting and weakness, foot deformity, and sometimes enlarged nerves on clinical examination (Ouvrier et al., 1987). The terminology used for the early‐onset demyelinating neuropathies can be unclear. The clinical and neurophysiologic phenotype of DSD includes forms of Charcot–Marie–Tooth disease (CMT) types 3 and 4 (Yiu & Ryan, 2012). Dominant *de novo* mutations in peripheral myelin protein 22 (*PMP22*; Roa, Dyck, Marks, Chance, & Lupski, 1993), myelin protein zero (*MPZ*; Hayasaka et al., 1993; Warner et al., 1998) or early growth response 2 (*EGR2*) are the most common causes of DSD. In more than 50% of cases, a causative genetic mutation cannot be identified (Baets et al., 2011).

High‐resolution peripheral nerve ultrasound allows rapid, noninvasive imaging of peripheral nerves (Goedee et al., 2013). Few dedicated pediatric nerve ultrasound studies have been published. Yiu et al. (2015) found a two‐ to threefold increase in nerve cross‐sectional area (CSA) in children with CMT1A compared to controls. Nerve ultrasound may play a role in the diagnosis of pediatric inherited neuropathies, and in the era of next‐generation sequencing, knowledge of specific nerve sonographic features, in addition to clinical and neurophysiologic findings, may assist in the interpretation of genetic testing results.

We evaluated nerve CSA in children with DSD in comparison both with age‐ and sex‐matched healthy controls and age‐matched children with CMT1A.

## 2 MATERIALS AND METHODS

### 2.1 Study objectives

The primary objective of this study was to investigate differences in nerve CSA in children with DSD as measured by peripheral nerve ultrasound compared to healthy controls. Given the more severe clinical course and neurophysiologic abnormalities in DSD compared to CMT1A, we hypothesized that nerve CSA would be significantly greater in children with DSD compared to their healthy controls, and greater than matched children with CMT1A. The secondary objective was to define the clinical features of DSD in this cohort.

### 2.2 Study design and recruitment

This cross‐sectional, matched, case–control study was conducted at The Royal Children's Hospital, Melbourne (RCH). Children with DSD were recruited from the neuromuscular clinic. Inclusion criteria for children with DSD included (1) symptom onset in the first 2 years of life; (2) delayed motor development; and (3) median nerve MNCV ≤ 12 m/s. Healthy controls were enrolled at a ratio of 1:1 and were age‐matched (within one year either side) and sex‐matched. They were recruited from friends and family members of RCH staff and were screened for symptoms and signs of neuromuscular disorders. Data from matched children with CMT1A from a previous study of nerve ultrasound (Yiu et al., 2015) were also used for comparison. For children with CMT1A, age match (within 1 year) was chosen in preference to sex match.

### 2.3 Nerve ultrasound

Nerve ultrasound images were obtained in the dominant upper and lower limb at the following sites: (1) median nerve at the midhumerus (two‐thirds from the lateral tip of the acromion to the lateral epicondyle of the humerus), at the elbow (in the cubital fossa), at the forearm (lower third), and at the wrist (at the level of the lunate); (2) ulnar nerve at the midhumerus, just distal to the elbow (one‐fifth forearm distance distal to the medial epicondyle), and at the midforearm; (3) tibial nerve at the ankle; and (4) sural nerve at the ankle.

All nerves were transversely imaged with the probe perpendicular to the nerve, providing the most accurate and smallest CSA. Nerve CSA was measured by tracing the nerve just inside the hyperechoic rim. Three images were taken and averaged at each nerve site. Ultrasound images were obtained using a General Electric (Fairfield, CT) BT12 LOGIQ‐e imaging system with a high‐frequency (8–18 MHz) hockey stick transducer. For upper limb nerves, participants were examined in the supine position with the arm supinated. For lower limb nerves, participants were examined in the left or right lateral position. The ultrasonographer (EY) was not blinded to the clinical status of the participants.

### 2.4 Clinical assessment

During the same study visit as the ultrasound assessment, weight and height were recorded and body mass index (BMI) was calculated. Children with DSD also underwent a neurologic assessment, including history, physical examination, and assessment using the Charcot–Marie–Tooth Pediatric scale (CMTPedS), a clinical rating tool designed to measure disease severity in children with CMT (Burns et al., 2012). This 11‐item rating tool provides a total score between 0 and 44, with a higher score reflecting greater impairment.

### 2.5 Statistical analyses

Statistical analysis was performed using Stata statistical software 13.0 (Stata Corp. 2013, College Station, TX, USA). Demographics, body metrics, and clinical features were summarized descriptively for all participants. Paired t tests were used to compare the mean difference in nerve CSA between children with DSD and their matched controls, and between matched children with CMT1A. Individual results were also plotted graphically. Sample size was convenience‐based.

### 2.6 Standard protocol approvals, registrations, and patient consents

This study was approved by the RCH Human Research and Ethics Committee (HREC number 34242). Informed consent was obtained for all participants.

## 3 RESULTS

Five children with DSD were enrolled—one boy and four girls, with a mean age of 7.8 (*SD* 3.9) years. Key clinical and genetic features are shown in Table 1. Case 5 has been previously reported (Yiu et al., 2017). Five age‐ and sex‐matched controls were recruited with a mean age of 8.2 (*SD* 4.0) years. The greatest difference in age between each child with DSD and their matched control was 9.8 months (case 5—control 9.8 months older than DSD case). There was no significant difference in height, weight, or body mass index (BMI) between children with DSD and controls.

**Table 1:** Clinical and genetic findings in five children with Dejerine–Sottas disease

Case number	1	2	3	4	5
Sex (M/F)	F	M	F	F	F
Age at visit (years)	11.8	2.0	9.6	5.7	9.8
Gene mutation	Unclassified^a^	*EGR2* p.G356K (htz)	Unclassified^b^	*PMP22* p.S72L (htz)	*MPZ* p.K130R (htz)
Age onset (months)	6	At birth	15	6	6
Presenting symptoms and signs					
Motor delay	Yes	Yes^c^	Yes	Yes	Yes
Hypotonia	Yes	Yes	No	Yes	Yes
Hip dysplasia (bilateral)	Yes	No	No	No	Yes
Reflexes	Normal	Reduced	Normal	Unknown	Absent
Poor suck	Yes	Yes	No	No	No
Neurophysiologic findings					
Age at study	3 years, 8 months	7 months	3 years, 10 months	2 years, 2 months	1 year, 1 month
Median nerve MNCV (m/s)	3.0	4.1	9.1	Absent response	12.0
Current symptoms and signs					
Independently ambulant	No	No	Yes	No	No
Wears AFO's	Yes	Yes	No	Yes	Yes
Foot deformity	Yes^d^	Yes^d^	No	Yes^d^	Yes^e^
Hand weakness	Yes	Yes	No	Yes	Yes
Bulbar dysfunction	Yes	Yes	No	Yes	No
Reflexes	Absent	Absent	Absent	Absent	Absent
Hip flexion (R/L)^f^	2+/3	NP	5/5	4+/4+	2+/0
Ankle dorsiflexion (R/L)^f^	0/0	NP	5−/5−	4/4	3/3+
Shoulder abduction (R/L)^f^	4/4	NP	5/5	4/4	0/0
Hand intrinsics (R/L)^f^	2/2	NP	4+/4+	4/4	0/0
CMTPedS score (0–44)^g^	42	NP	12	30	40
Complications					
Respiratory insufficiency	Yes	Yes	No	No	Yes
Scoliosis	Yes	Yes	No	Yes	Yes
Hip dysplasia (bilateral)	Yes	No	No	Yes	Yes

AFO, ankle‐foot orthoses; CMTPedS, Charcot–Marie–Tooth disease Pediatric Scale; htz, heterozygous mutation; MNCV, motor nerve conduction velocity; NP, not performed.

a Heterozygous deletion of exons 4 and 5 of *PMP22* detected with no mutation in other *PMP22* allele.

b Heterozygous p.Q203X mutation in *PRX* detected, with no mutation in other *PRX* allele.

c Presented at birth with hypotonia but also documented to have motor delay in first 2 years of life.

d Pes planus.

e Equinovarus foot and ankle deformity.

f MRC (Medical Research Council) score.

g The CMTPedS provides a total score between 0 and 44. A higher score reflects greater impairment. The scale is validated for children >3 years of age.

Clinical features of the five children with DSD are summarized in Table 1. The mean age at onset was 6.6 (*SD* 5.4) months. All had delayed motor development. Four children presented with delayed motor milestones, hypotonia, and/or hip dysplasia. The fifth child (case 2) presented at birth with hypotonia, hyporeflexia, aspiration, and feeding problems and also had motor delay in the first two years of life. At presentation, three had hyporeflexia or areflexia.

At the time of the study, all children had gait abnormalities. Four were unable to walk or stand independently, wore ankle‐foot orthoses (AFO's), and had foot deformity and weakness of the hand muscles. The fifth child was independently ambulant but had frequent trips and falls. Orthopedic complications were common—four children had scoliosis and three bilateral hip dysplasia. Three children had respiratory insufficiency, with reduced vital capacity on spirometry and/or recurrent respiratory infections requiring hospital admissions. One child received nocturnal noninvasive ventilation (case 1). Three children had bulbar dysfunction.

All children had distal lower limb muscle wasting—two mild, two moderate, and one with severe wasting extending above the knee. Wasting of the thenar, hypothenar, and intrinsic hand muscles was moderate in one child and severe in two. Weakness was most marked distally; however, most children also had proximal muscle weakness of both upper and lower limbs. The CMTPedS scores reflect the overall severity of the neuropathy, with a mean of 31.0 (*SD* 11.8).

### 3.1 Neurophysiologic, neuropathologic, and genetic findings

Nerve conduction studies were performed in all children before four years of age. Median nerve MNCV was 12 m/s or less in all cases (Table 1).

One child (case 5) had a sural nerve biopsy at 15 months of age, which showed a severe depletion of large diameter myelinated fibers, thinly myelinated intermediate diameter axons, and absent onion bulbs. Focal myelin folding was present on electron microscopy, a finding well described in *MPZ*‐related neuropathies (Yiu et al., 2017).

Three children have a definitive genetic diagnosis, with mutations in different genes (Table 1). Cases 4 and 5 have previously reported pathogenic mutations in *PMP22* (p.Ser72Leu) and *MPZ* (p.Lys130Arg), respectively (Gabreels‐Festen et al., 1996; Roa et al., 1993). Case 2 has a novel p. Gly356Lys de novo mutation in *EGR2*, considered likely to be pathogenic [disease‐causing on in silico analysis, low frequency in ExAC (1/121266), and proximity to other reported mutations p.Asp355Val and p.Arg359Trp]. Case 3 had a relatively mild clinical phenotype but marked conduction slowing. She is heterozygous for a novel nonsense mutation in *PRX* (p.Gln203*); a second mutation in this gene or other genes associated with CMT has not been identified on whole‐exome sequencing. Case 1 is heterozygous for a deletion of exons 4 and 5 of *PMP22* in one allele, without a demonstrable mutation or copy number variant on the other allele, or mutations in any other genes associated with CMT, when tested on a neuromuscular gene chip. We assume there may be another, at present, unidentified gene mutation contributing to her DSD phenotype. Coverage of the coding regions of *PRX* (case 3) and *PMP22* (case 1) was effectively complete.

### 3.2 Comparison of nerve CSA between children with DSD, healthy controls, and children with CMT1A

**Figure 1:**
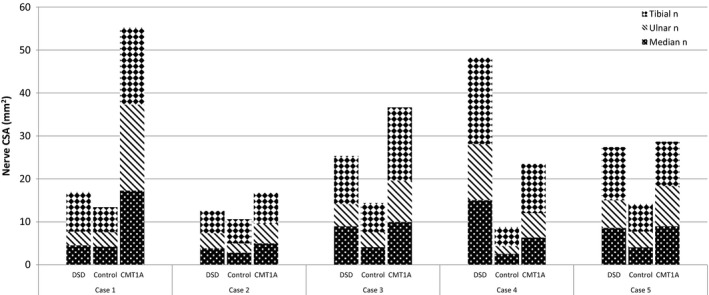
Nerve CSA in children with DSD compared to matched controls and children with CMT1A. Nerve CSA at three sites shown for each child with DSD compared to their matched control and CMT1A case. Each stacked column shows nerve CSA for the median nerve at the forearm (shaded black with white spots), ulnar nerve at the forearm (black and white stripes), and tibial nerve at the ankle (black and white diamonds). CMT1A, Charcot–Marie–Tooth disease type 1A; CSA, cross‐sectional area; DSD, Dejerine–Sottas disease

Whilst nerve CSA was greater in children with DSD, there was no significant difference in nerve CSA between children with DSD and healthy controls at any site when analyzed using paired t tests (Table 2). Due to the genetic heterogeneity and small size of the cohort, nerve CSA was also individually compared between each child with DSD and their control. The difference in nerve size between children with DSD and controls differed markedly between participants. Nerve CSA was similar to matched controls in cases 1 and 2. Nerve CSA was markedly increased in cases 4 (*PMP22* point mutation) and 5 (*MPZ* point mutation) compared to control values in all nerves studied. Case 3 showed variable increase in nerve CSA between sites, with enlargement seen mainly in the median and tibial nerves compared to the matched control (Figure 1 and Table 3).

**Table 2:** Nerve cross‐sectional area in five children with Dejerine–Sottas disease compared to controls

Nerve CSA, mm^2^ Measurement site	DSD, mean (*SD*)	Controls, mean (*SD*)	Mean difference (95% CI)	*p*‐value^a^
Median nerve at wrist	8.1 (3.6)	4.8 (0.6)	3.3 (−1.3, 7.9)	.11
Median nerve at forearm	8.2 (4.5)	3.6 (0.8)	4.6 (−1.4, 10.6)	.10
Median nerve at elbow^b^	10.9 (6.5)	4.3 (1.3)	6.6 (−12.2, 25.3)	.27
Median nerve at midhumerus^c^	15.5 (2.5)	3.7 (0.4)	11.9 (−14.4, 38.1)	.11
Ulnar nerve at forearm	6.3 (4.0)	2.9 (0.8)	3.4 (−2.2, 9.0)	.17
Ulnar nerve at elbow	5.8 (3.2)	2.8 (0.8)	3.0 (−1.4, 7.4)	.13
Ulnar nerve at midhumerus^d^	8.1 (4.3)	3.0 (0.7)	5.2 (−2.3, 12.6)	.11
Tibial nerve at ankle	11.6 (5.5)	5.8 (0.9)	5.8 (−1.7, 13.3)	.10
Sural nerve at ankle^d^	1.9 (0.4)	1.4 (0.2)	0.5 (−0.4, 1.5)	.18

CSA, cross‐sectional area; *SD*, standard deviation; CI, confidence interval.

a *p*‐value from paired t test.

b Data available on three children from each group.

c Data available on two children from each group.

d Data available on four children from each group.

**Table 3:** Nerve cross‐sectional area in children with DSD compared to matched controls and children with CMT1A

Nerve	Site	Case	Nerve CSA (mm^2^)		
			DSD	Control	CMT1A
Median nerve	Wrist	1	5.9	5.4	8.8
		2	3.9	4.0	5.1
		3	8.5	4.9	9.6
		4	13.3	4.2	8.2
		5	8.8	5.3	7.5
	Forearm	1	4.6	4.3	17.3
		2	3.9	2.8	5.1
		3	9.0	4.1	10.0
		4	15.1	2.6	6.4
		5	8.6	4.1	9.0
	Elbow	1	4.7	4.8	NP
		2	NP	3.5	NP
		3	7.0	NP	NP
		4	17.7	2.9	NP
		5	10.3	5.3	NP
	Midhumerus	1	NP	5.0	NP
		2	NP	4.1	NP
		3	10.8	NP	NP
		4	17.3	3.4	NP
		5	13.8	4.0	NP
Ulnar nerve	Forearm	1	3.2	3.3	20.0
		2	3.5	2.2	4.6
		3	5.1	3.5	9.6
		4	13.1	1.8	5.7
		5	6.4	3.7	9.4
	Elbow	1	3.8	3.0	12.6
		2	3.1	2.1	4.6
		3	4.3	3.9	12.3
		4	10.8	2.0	4.5
		5	7.1	3.2	8.6
	Midhumerus	1	5.2	3.9	NP
		2	3.7	2.7	NP
		3	4.8	NP	NP
		4	12.5	2.2	NP
		5	11.1	3.0	NP
Tibial nerve	Ankle	1	9.2	5.8	17.9
		2	5.2	5.6	7.2
		3	11.2	6.8	17.0
		4	20.2	4.4	11.4
		5	12.4	6.5	10.3
Sural nerve	Ankle	1	1.6	1.6	4.6
		2	NP	1.2	NP
		3	1.6	1.2	2.4
		4	2.5	1.1	1.7
		5	1.8	1.5	5.1

CMT1A, Charcot–Marie–Tooth disease type 1A; CSA, cross‐sectional area; DSD, Dejerine–Sottas disease; NP, not performed.

An additional comparison of nerve CSA in DSD was made with age‐matched children with CMT1A from a previous study (Yiu et al., 2015). There was less than 7 months difference in age between each child with DSD and their matched CMT1A subject. Three children were not sex‐matched. There was no significant difference between height or weight between children with DSD and CMT1A (data not shown). Figure 1 and Table 3 also show comparative nerve size in children with CMT1A. Case 4 (*PMP22* point mutation) demonstrated the greatest degree of nerve enlargement, with nerve CSA values approximately twice as large compared to a matched case of CMT1A and five times as large compared to a matched healthy control. Nerve size in cases 3 and 5 was enlarged to a similar degree to that seen in CMT1A.

## 4 DISCUSSION

This single‐center study confirms the genetic heterogeneity of the early‐onset demyelinating neuropathies of childhood, is the first to describe the high‐resolution nerve ultrasound features of this cohort of children, and demonstrates a marked variability in the degree of nerve enlargement in DSD. The latter likely reflects the genetic heterogeneity of DSD and subsequent downstream pathobiologic processes.

We previously showed a marked (two‐ to threefold) and widespread increase in nerve CSA in a genetically homogeneous cohort of children with CMT1A (Yiu et al., 2015). The present study sought to compare nerve CSA in children with DSD to controls, and to children with CMT1A, with the hypothesis that the degree of nerve enlargement in DSD would exceed that of CMT1A, given its generally more severe clinical and neurophysiologic phenotype.

Our hypothesis was disproved; we found marked variability in the degree of nerve enlargement in children with DSD compared to age‐ and gender‐matched healthy controls. Case 4 was the only subject in our cohort with findings in keeping with our original hypothesis. She demonstrated the greatest increase in nerve CSA—four to five times greater than her matched control and approximately twice that of an age‐matched child with CMT1A. Interestingly, she is heterozygous for a point mutation in *PMP22*, the same gene duplicated in CMT1A.

Two other children demonstrated significant nerve enlargement. The first, a child with a heterozygous point mutation in *MPZ* had nerve CSA approximately double that of her matched healthy control. The second child (case 3) showed variable nerve enlargement at different sites. It is not clear if this finding represents true variability in nerve enlargement in this case or sampling error. The genetic etiology of this case remains unclear, as a second mutation has not been found in the *PRX* gene. The 2‐year‐old boy with an *EGR2* mutation showed minimal to no nerve enlargement, and the remaining child (case 1) showed no increase in nerve CSA. The genetic cause of this latter case is unclear. She has a single exonic deletion in one *PMP22* allele but no identifiable mutation in the other allele. This is not thought to explain her clinical phenotype—heterozygous *PMP22* deletions are usually associated with hereditary liability to pressure palsies, whilst children with early‐onset demyelinating neuropathies usually have compound heterozygous deletions of *PMP22* (Abe et al., 2010; Al‐Thihli et al., 2008). Given the lack of nerve enlargement in this case compared to the child with a *PMP22* point mutation, one could hypothesize that this child either has a mutation in another gene or, if she does have a second, as yet undetected mutation in *PMP22*, that this reflects mutation‐specific effects of different *PMP22* mutations on nerve enlargement.

The increase in nerve CSA seen on nerve ultrasound parallels the increase in total transverse fascicular area (TTFA) seen in neuropathologic studies of CMT. In CMT1A, TTFA is 1.5‐ to twofold that of healthy controls (Gabreels‐Festen, 2002), reflecting the increase in nerve CSA seen by nerve ultrasound (Yiu et al., 2015). Similarly, Gabreels–Festen documented a TTFA/normal ratio of 393%–458% in children with *PMP22* missense mutations and a 92%–276% in individuals with *MPZ* missense mutations; Gabreels‐Festen (2002) findings in keeping with the changes seen in nerve CSA in the present study. The marked increase in TTFA is thought to be secondary to an increase in collagen fibers and endoneurial extracellular matrix (Gabreels‐Festen, 2002; Yiu et al., 2015). Large numbers of onion bulbs may also contribute to increased fascicular size in DSD. Unfortunately, there is little published data on TTFA or endoneurial collagen in patients with *EGR2* or *PRX* mutations. Only one child in this study (with an *MPZ* mutation) had a nerve biopsy performed. This biopsy showed a severe reduction in myelinated fibers, thin myelin sheaths, and an absence of onion bulbs; features not expected to cause significant nerve enlargement. Unfortunately, TTFA and endoneurial collagen content were not reported. This, along with the fact that the biopsy was performed at 15 months of age, 8 years prior to the nerve ultrasound, makes neuropathologic and sonographic correlations difficult.

The variable nerve ultrasound findings in this study reflect the genetic heterogeneity of DSD, confirming that the underlying pathobiologic processes and downstream morphological abnormalities vary between DSD subtypes. This has been demonstrated at a neuropathologic level—where findings such as loss of myelinated fibers, reduced myelin thickness, and a marked increase in onion bulb formations are common to all forms of DSD, but additional pathological findings, such as focal myelin folding, are associated with specific genetic subtypes. If we postulate that the main contributor to nerve enlargement is increased volume of endoneurial extracellular matrix and perhaps onion bulb formations, the variable nerve ultrasound findings in this study may reflect the effect of different gene mutations on extracellular matrix production and onion bulb formation, either directly or via abnormal Schwann cell‐extracellular matrix interplay (Colognato & Tzvetanova, 2011; Yiu et al., 2015).

In CMT1A, nerve CSA shows a modest correlation with disability (Yiu et al., 2015). Although limited by the small sample size, the degree of nerve enlargement did not appear to correlate with clinical severity in this study, as measured by the CMTPedS total score. Four children had marked neurologic disability; three of the four children old enough to perform the CMTPedS had scores greater than 30, indicating severe impairment.

This study has a number of limitations. Due to low prevalence of DSD, the sample size was small, limiting statistical analysis. The ultrasonographer was not blinded to the clinical status of the patient.

The workup of early‐onset neuropathies involves clinical history and examination, neurophysiologic studies, and genetic testing. In the era of next‐generation sequencing, specific nerve sonographic features may act as an adjunct to clinical and neurophysiologic phenotyping of DSD, and hence, interpretation of genetic testing results particularly in the interpretation of variants of unknown significance. The rapid and noninvasive nature of nerve ultrasound makes it an attractive imaging technique in children, complementing neurophysiologic testing. Studies in larger DSD cohorts, especially those with similar genetic etiology, are required to confirm the findings of the current study and determine whether nerve ultrasound can accurately differentiate between different genetic causes of DSD. Larger cohorts may show correlation with disease severity in genetically homogeneous cohorts of patients. Longitudinal studies will provide insight into the responsiveness of nerve ultrasound to change over time, and its value as a biomarker in natural history studies and trials of future therapies.

## CONFLICT OF INTEREST

None declared.
